# Early inflammatory markers as prognostic indicators following allogeneic stem cell transplantation

**DOI:** 10.3389/fimmu.2023.1332777

**Published:** 2024-01-03

**Authors:** Kriti Verma, Wayne Croft, David Greenwood, Christine Stephens, Ram Malladi, Jane Nunnick, Jianmin Zuo, Francesca A. M. Kinsella, Paul Moss

**Affiliations:** ^1^Institute of Immunology and Immunotherapy, University of Birmingham, Birmingham, United Kingdom; ^2^Centre for Computational Biology, University of Birmingham, Birmingham, United Kingdom; ^3^Centre for Clinical Haematology, Queen Elizabeth Hospital, Birmingham, United Kingdom

**Keywords:** stem cell transplantation, prognostic factors, GvHD, relapse, inflammation

## Abstract

Allogeneic stem cell transplantation is used widely in the treatment of hematopoietic malignancy although graft versus host disease and relapse remain major complications. We measured the serum protein expression of 92 inflammation-related markers from 49 patients at Day 0 (D0) and 154 patients at Day 14 (D14) following transplantation and related values to subsequent clinical outcomes. Low levels of 7 proteins at D0 were linked to GvHD whilst high levels of 7 proteins were associated with relapse. The concentration of 38 proteins increased over 14 days and higher inflammatory response at D14 was strongly correlated with patient age. A marked increment in protein concentration during this period associated with GvHD but reduced risk of disease relapse, indicating a link with alloreactive immunity. In contrast, patients who demonstrated low dynamic elevation of inflammatory markers during the first 14 days were at increased risk of subsequent disease relapse. Multivariate time-to-event analysis revealed that high CCL23 at D14 was associative of AGvHD, CXCL10 with reduced rate of relapse, and high PD-L1 with reduced overall survival. This work identifies a dynamic pattern of inflammatory biomarkers in the very early post-transplantation period and reveals early protein markers that may help to guide patient management.

## Introduction

Allogeneic hemopoietic stem cell transplantation (allo-HSCT) is a highly effective therapy for many patients with haematological malignancy. However, allo-HSCT remains associated with morbidity and mortality from complications such as graft versus host disease and disease relapse. As such there is substantial interest in identifying approaches that can help to predict individual patient outcomes and guide appropriate management in the post-transplant period.

The utility of such approaches would be most beneficial if they were available in the very early period of transplantation although this may appear challenging as many transplant-related complications occur several months or years following the procedure. However, many lines of evidence indicate that immunological determinants of long-term transplant outcome are established in the very early post-transplant period. Examples of this include the efficacy of cyclophosphamide administration at day 4+ and the impact of relative concentration of immune suppression within the first three weeks post-transplant (1). Indeed, alloreactive T cells are identifiable within the first two weeks following stem cell infusion and serum levels of ST2 and REG3α as early as day 7 are associated with risk of GvHD (2). As such it is likely that the early kinetics and intensity of the donor alloreactive immune response are key determinants of subsequent risk of clinical complications such as GvHD or relapse. Assessment of immune parameters within this early period could provide valuable prognostic information and may help to stratify treatment protocols.

A range of studies have measured serum protein concentrations to assess their prognostic value in determining patient outcome (3). These have proven of particular interest in relation to risk of acute GvHD where ST2 has been positively correlated with incidence in several studies, a relationship that is seen as early as day 7 or day 14 post-transplant (2, 4, 5). Early elevation of IL-6 levels has also been linked to acute GvHD (6) whilst REG3a and Tim-3 have also been linked to gastrointestinal inflammation (5, 7, 8). Importantly, the clinical importance of proteomic biomarkers can extend beyond prognostic value as antibody-mediated blockade of ST2 or IL-6 has shown value in reducing the incidence of subsequent acute GvHD (9). Concentration of markers such as ST2, CXCL9, CXCL10, CD163 MMP-3 and osteopontin have also shown predictive value in the development of chronic GvHD although these assessments are typically assessed at later time points (10–12).

Here we determined serum concentration of 92 inflammation-related proteins in patients at day 0 and day 14 following transplantation. We hypothesize that monitoring the levels of inflammatory proteins on the day of transplant as a baseline and comparing them to levels two weeks post-transplant can serve as a valuable indicator of the inflammatory status in patients undergoing stem cell transplantation. By assessing the inflammatory protein concentrations at both day 0 and day 14, we aim to uncover the temporal patterns and trajectories that may be more informative than absolute values at individual time points.

## Methods

### Patient cohort

Patients who were undergoing allogeneic stem cell transplantation for management of haematopoietic malignancy were eligible for study and were recruited prospectively between 2015 and 2019. Patients with GVHD symptoms within 14 days of transplantation were excluded from analysis. Serum samples taken on the day of transplantation (49 patients, Day-0 cohort) and at 14 days post-transplant (49 matched samples and an additional 105 patients; Day-14 cohort). Demographic data included age and gender of both patient and donor, CD34+ cell dose, underlying diagnosis (lymphoid or myeloid), HLA mismatch status, hematopoietic cell transplantation-specific comorbidity index (HCT-CI; 0, 1, 2, or 3+), conditioning regimen (total body irradiation (TBI) and reduced intensity conditioning (RIC), serotherapy (anti-thymocyte globulin (ATG) or alemtuzumab (Campath)), GvHD prophylaxis (ciclosporin or ciclosporin & mycophenolate or methotrexate (combination)) and patient cytomegalovirus status. Follow up information included time and type of event following SCT (AGvHD, CGvHD, relapse, death) as well as the AGvHD grade.

### Proteomic analysis

Protein concentration was measured in serum using the OLINK inflammation panel (Olink Proteomics AB, Uppsala, Sweden) (13) which includes 92 protein targets. Assay readout is measured as Normalized Protein eXpression (NPX) in arbitrary units on a log2 scale.

### Differential protein expression

Serum NPX data were imported and analysed using R version 4.0.2. (R Core Team 2020). For Day-14 vs Day-0 comparisons, paired samples Wilcoxon tests were applied to data from 49 paired D0-D14 samples. Assessment of differential protein expression between clinical outcomes was assessed by Mann-Whitney U test with correction for multiple testing using the Benjamini Hochberg method to control for false discovery rate (14). For volcano plot visualisation of test results, fold change estimates (the *estimate* value from test output) are used, which are calculated as median of the differences in sample from x and sample from y. Correlations of protein expression level with patient clinical characteristics were calculated using the R function rcorr using spearman rank method. Scatter plots of NPX stratified by day or clinical outcome and volcano plots to visualise results of statistical tests were generated using ggplot2 (15).

Linear dimensionality reduction was performed by PCA and visualised with factor loadings using the factoextra R package (16). Nonlinear dimensionality reduction was via applying the Uniform Manifold Approximation and Projection (UMAP) method. Heatmaps were generated with complexHeatmap (17).

Absolute protein concentrations in serum were determined using ELISA kits from Abcam (MA, USA) for CDCP1 (ab253216), LIFR (ab213806), PD-L1 (ab277712), Flt3L (ab213780), CCL23 (ab100611) and IL-6(A78324) using manufacturer’s protocols. The protein concentrations that were identified as associative of clinical outcome at day 14 (PD-L1, LIFR, Flt3L, CCL23, IL-6 and CDCP1) were related to Olink NPX values in order to support future clinical application in clinical prognosis (**Supplementary Figure S3**).

### Survival analysis

Survival analysis was conducted on the Day-14 cohort. Subjects with missing outcome information were excluded (N=9). Survival events were studied with the Kaplan–Meier (KM) estimator and Cox proportional hazards (Cox PH) models, using the Survival R package (18). Competing events were studied with cumulative incidence function (CIF) estimates and Fine-Gray (FG) models using the “cmprsk” R package (19, 20).

The primary survival endpoints were the time to first-event onset (GvHD-free, relapse-free survival (GRFS)) and the time to death attributable to any cause, overall survival (OS). GRFS events included the onset of AGvHD (grade II-IV), CGvHD, relapse and death. The primary competing events investigated were the time to non-relapse mortality (NRM) and the time to relapse-related mortality (RM). NRM was defined as death without prior relapse, and RM as death preceded by relapse. Secondary endpoints investigated included the onset of: relapse, AGvHD (grade II-IV) and CGvHD. For RM and relapse, NRM was modelled as the competing event. Whereas for AGvHD and CGvHD, the competing event was death attributable to any other cause.

Survival analysis was restricted to only the serum proteins identified as differentially expressed when stratified by outcome to reduce the rate of type I errors. NPX values were first standardised to have a mean of 0 and SD of 1. Variable selection was conducted for the primary events with the least absolute shrinkage and selection operator (LASSO) (21, 22). Variables which violated the proportional hazards (PH) assumption were excluded; a PH test based on Schoenfeld residuals was used for CoxPH models (23) and a PH test based on the cumulative sum of residuals was used for Fine-Gray models (24).

Missing observations in the clinical variables were quantified (**Table 1**) and imputed from the observed data by predictive mean matching (for continuous variables), logistic regression (for binary variables) and polytomous regression (for ordinal variables) (25, 26).. Multivariate imputation by chained equations (MICE) (27) was applied with fully conditional specification to produce 5 imputations of the missing observations. Models were fitted to each imputation and coefficients were pooled into a single estimate by applying Rubin’s rules (28).

**Table 1:** Patient characteristics.

Variable	Group	Day-0 cohort		Day-14 cohort	
		Overall	Missing (%)	Overall	Missing (%)
**N**		49		154	
**Age at transplant (median [IQR])**		52.0 [43.0, 61.0]	0	52.0 [42.8, 61.0]	1.3
**CD34+ dose (median [IQR])**		5.2 [4.2, 5.6]	4.1	5.1 [4.3, 6.0]	4.5
**CMV status (%)**	Negative	16 (32.7)	0	49 (32.5)	1.9
	Positive	33 (67.3)		102 (67.5)	
**Diagnosis (%)**	Lymphoid	14 (28.6)	0	47 (30.9)	1.3
	Myeloid	35 (71.4)		105 (69.1)	
**Donor age (median [IQR])**		33.0 [26.0, 47.0]	4.1	33.0 [25.0, 47.2]	37.7
**GvHD prophylaxis (%)**	Ciclosporin	36 (73.5)	0	102 (67.1)	1.3
	Combination	13 (26.5)		50 (32.9)	
**HLA mismatch status (%)**	No	44 (89.8)	0	104 (88.9)	24
	Yes	5 (10.2)		13 (11.1)	
**Patient sex (%)**	Female	12 (24.5)	0	56 (36.8)	1.3
	Male	37 (75.5)		96 (63.2)	
**RIC (%)**	No	20 (40.8)	0	65 (42.8)	1.3
	Yes	29 (59.2)		87 (57.2)	
**Sex matched donor (%)**	No	18 (36.7)	0	67 (44.7)	2.6
	Yes	31 (63.3)		83 (55.3)	
**HCT-CI (%)**	0	14 (28.6)	0	48 (41.0)	24
	1	5 (10.2)		14 (12.0)	
	2	14 (28.6)		27 (23.1)	
	3+	16 (32.7)		28 (23.9)	
**Serotherapy (%)**	ATG	21 (42.9)	0	51 (33.6)	1.3
	Campath	25 (51.0)		84 (55.3)	
	None	3 (6.1)		17 (11.2)	
**TBI (%)**	No	41 (83.7)	0	123 (80.9)	1.3
	Yes	8 (16.3)		29 (19.1)	
**AGVHD (%)**	Censored	30 (62.5)	2	88 (59.1)	3.2
	Event	18 (37.5)		61 (40.9)	
**CGVHD (%)**	Censored	39 (81.2)	2	122 (81.3)	2.6
	Event	9 (18.8)		28 (18.7)	
**Relapse (%)**	Censored	36 (73.5)	0	105 (70.5)	3.2
	Event	13 (26.5)		44 (29.5)	
**Death (%)**	Censored	36 (73.5)	0	100 (65.8)	1.3
	Event	13 (26.5)		52 (34.2)	

Patient characteristics and transplant outcomes depicted in the D0 and D14 cohorts with percentage missing variables.

## Results

### Low inflammatory protein levels on the day of transplantation associate with GvHD whilst higher levels are seen in patients who subsequently relapse

**Figure 1:**
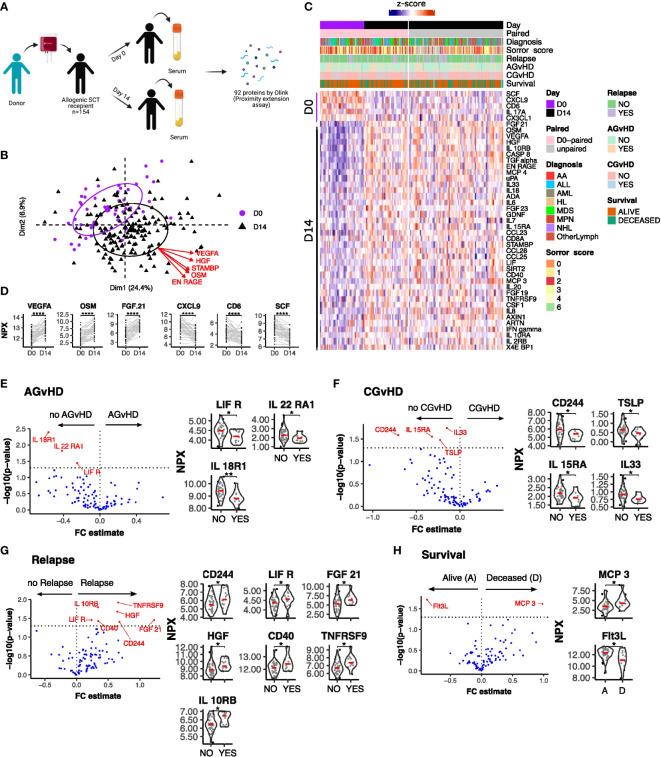
Serum protein expression on the day of stem cell infusion is related to subsequent clinical outcome. **(A)** Schematic representation of study. **(B)** PCA plot of protein serum concentration at Day 0 and D 14. Arrows indicate factor loadings for the top 5 proteins ranked by variance. **(C)** Hierarchical clustering of proteins whose concentration was different between day 0 and day 14 (p<0.01, paired samples Wilcoxon test. **(D)** Example of kinetics of expression of 3 proteins which showed the most marked increase or decrease in expression between D0 and D14. Ranking by p value. Univariate testing for differential expression of Day 0 serum protein levels by clinical outcomes: **(E)** Acute GvHD: Yes (13) vs No (35); **(F)** Chronic GvHD: Yes (9) vs No (39); **(G)** Relapse: Yes (13) vs No (36) **(H)** Survival: Deceased **(D)** (13) vs Alive **(A)** (36). Proteins labelled in red showed a significant difference (p<0.05) by Man Whitney after Benjamini-Hochberg (BH) multiple testing correction. FC estimate is calculated as -1*(median difference between samples from eac distribution). Violin plots of Normalised Protein eXpression (NPX) are shown for the proteins found to have significant associations for each clinical outcome. (BH corrected p < 0.0001****; p ≤ 0.01**; p ≤ 0.05*).

Serum concentrations of 92 inflammation-related proteins were measured at day 0 and day 14 following stem cell transplantation from 49 patients (**Figure 1A**). An additional analysis was performed on 105 patients at day 14 alone. Principal component analysis (PCA) and expression profiles of day-specific signatures showed that protein concentrations varied substantially between day 0 and day 14 (**Figures 1B–D**).

Initial assessment studied how protein concentrations on day 0 were related to clinical outcome (**Figures 1E–H**). Differential expression analysis showed that low levels of IL18-R1, LIF-R and IL22-RA1 were seen in patients who subsequently developed acute graft versus host disease (AGvHD) (**Figure 1E**) whilst lower levels of CD244, TSLP, IL15-RA and IL-33 were observed in patients who developed chronic GvHD (**Figure 1F**). In contrast, increased concentrations of seven inflammatory proteins, CD244, LIF-R, FGF21, IL10-RB, HGF, CD40 and TNFRSF9, were associated with subsequent disease relapse (**Figure 1G**). Of note, values for LIF-R and CD244 (SLAMF4) were reciprocally correlated with risk of GvHD or relapse. Overall survival was higher in patients with elevated levels of the hemopoietic regulator Flt3 ligand (Flt3L) at the time of transplant. Flt3L is a reciprocal biomarker of progenitor cell mass (29) and as such these elevated levels may reflect superior pre-transplant conditioning. Low concentrations of the monocyte chemotactic protein CCL7 (MCP3) were also seen in patients with reduced overall survival although neither of these proteins were associated with other clinical features (**Figure 1H**).

**Figure 2:**
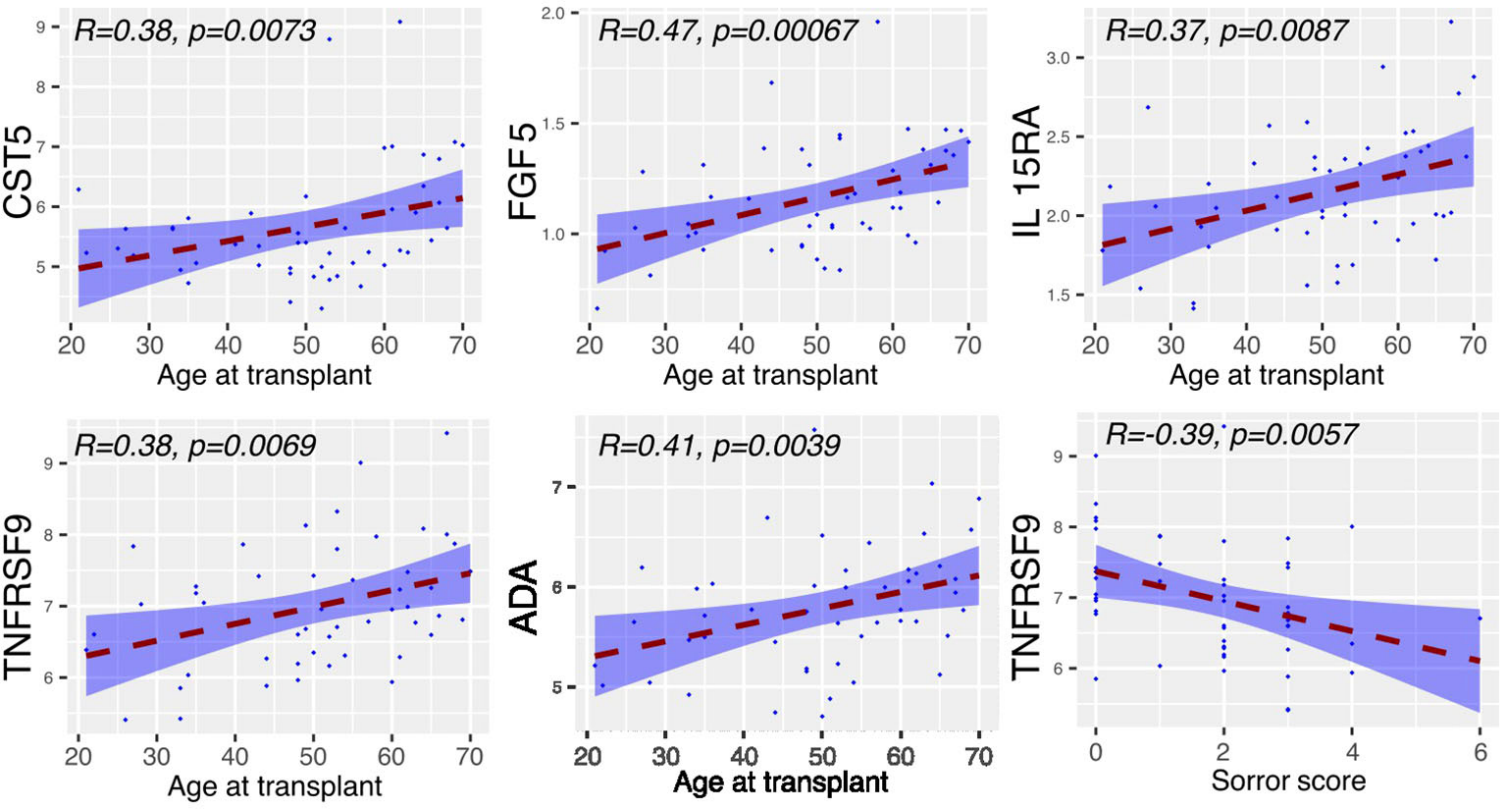
Serum proteins at Day 0 displaying correlation with clinical covariates. Relative concentration of serum proteins is shown on the y-axis and correlated with patient age, mononuclear cell dose, Sorror score and T cell dose. Significant protein-clinical variable correlations are shown with absolute correlation coefficient (R) > 0.2 (p<0.01). Dashed line represents the linear model fit to the data points with shading for 95% confidence interval.

The concentration of inflammatory proteins at day 0 was then related to patient demographics and transplant variables (n=49) (**Figure 2**). The concentration of 5 proteins, CST5, FGF5, IL15-RA, TNFRSF9 and ADA, correlated positively with patient age whilst TNFRSF9 values fell with an increasing Sorror score.

These findings indicate that lower levels of inflammatory proteins at baseline were associated with increased risk of GvHD and protection from relapse, both features of a strong alloreactive immune response.

### Inflammatory markers are markedly increased in older patients at 2 weeks post transplant

**Figure 3:**
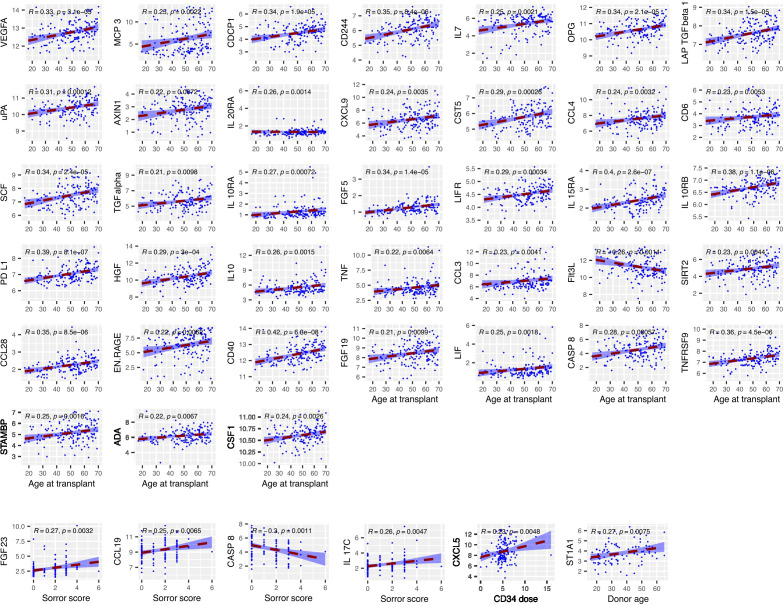
The concentration of several serum proteins is markedly elevated at day 14 in older patients. Relative concentration of serum proteins is shown on the y-axis and correlated with patient or donor age, CD34+ cell dose, mononuclear cell dose and Sorror score. Significant protein-clinical variable correlations are shown with absolute correlation coefficient (R) > 0.2 (p<0.01). Dashed line represents the linear model fit to the data points with shading for 95% confidence interval.

We next determined the concentration of inflammatory proteins at day 14 post-transplant and assessed how these were influenced by patient or donor age, CD34+ cell dose, mononuclear cell dose and Sorror score (n=154) (**Figure 3**). Of note, high expression of 37 proteins at day 14 was correlated with increasing patient age. This association was particularly strong for proteins such as CD40, IL-15RA, PD-L1 and IL-10RB. Indeed, Flt3-L was the only protein whose serum concentration was lower in older patients at this time point. In contrast, little impact of donor age was seen and this correlated only with higher concentration of the detoxifying sulfotransferase ST1A1.

As such these findings show that the age of the patient is a major determinant of the concentration of inflammatory markers in the early post-transplant period.

### Relative change of protein concentration between day 0 and day 14 associates with clinical outcomes

**Figure 4:**
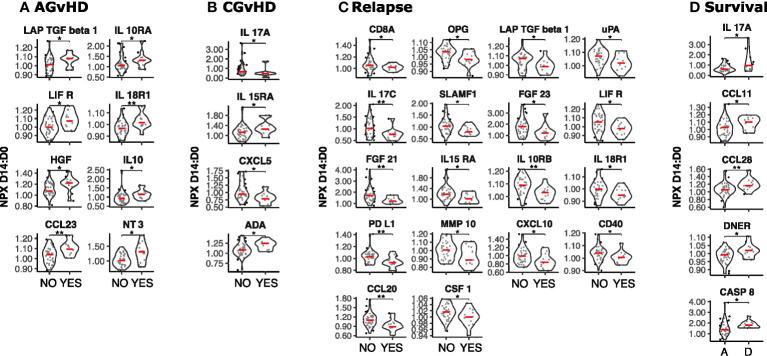
Temporal dynamics of serum protein concentration in the first two weeks post-SCT and its association with clinical outcome. The D14 to D0 ratio (D14:D0) of serum protein NPX values was determined and stratified by clinical outcome (n=49). **(A)** Acute GvHD: Yes vs No; **(B)** Chronic GvHD: Yes vs No; **(C)** Relapse: Yes vs No; **(D)** Survival: Deceased **(D)** vs Alive **(A)**. Significant differences in D14:D0 determined by Man Whitney after Benjamini-Hochberg (BH) multiple testing correction (BH corrected p≤0.01**; p≤0.05*).

In addition to absolute value it is also possible that the relative change of serum protein concentration within the first 2 weeks following transplantation might act as a determinant of subsequent clinical course. As such, we next determined the D14:D0 expression ratio for patients with paired values and related this to outcome (n=49) (**Figure 4**). The inflammatory environment during the post-transplant period was revealed by an increase in concentration of 38 proteins within the first 14 days compared to a decrease in only 5 (adjusted p < 0.01) (**Figure 1C**). The most marked increases were observed for VEGF-A, FGF21 and the cell growth regulator OSM (**Figure 1D**) whilst those falling included CD6, CXCL9 and the stem cell factor protein SCF.

High relative increase in 8 proteins was associated with increased risk of acute GvHD (**Figure 4A**). The strongest associations were seen for the T cell attractant chemokine CCL23 as well as IL18-R1 and it is notable that low levels of IL-18R1 at day 0 had also been associated with this risk (**Figure 1E**). Associations with CGvHD were more heterogeneous with both increased (IL-15RA, ADA) and decreased (IL-17A, CXCL5) ratios seen as correlates (**Figure 4B**). Again, low baseline levels of IL15RA at day 0 had also been linked with CGvHD (**Figure 1F**). The D14:D0 ratio of 18 proteins was also associated with risk of disease relapse but, strikingly, this was associated with a decrease, or relatively suppressed increment, for every protein studied. Key determinants included a range of major immunoregulatory proteins including PD-L1, CXCL10, and CCL20 (**Figure 4C**).

High relative change in 5 proteins (IL17A, CCL11, CCL28, DNER and CASP8) was associated with increased mortality (p ≤ 0.05) (**Figure 4D**).

These data show that the relative rate of change in protein concentration within the first two weeks post-transplant is strongly associated with subsequent outcome with high kinetic increase associating with AGvHD whilst decreased or muted responses are linked with subsequent disease relapse.

### High inflammatory protein expression at Day 14 associates with GvHD while low levels with disease relapse

**Figure 5:**
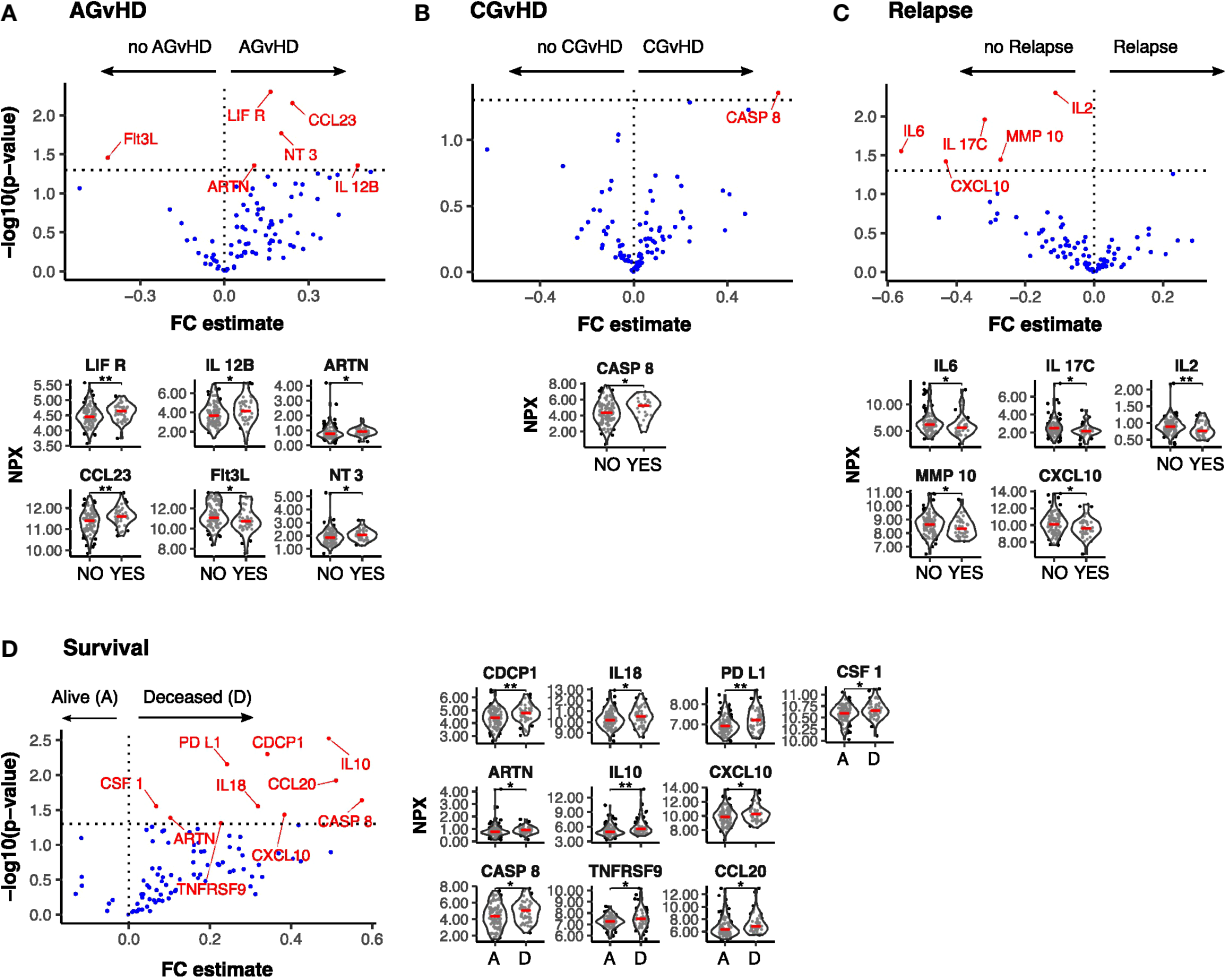
Differentially expressed serum protein markers at Day-14 post SCT by clinical outcome. Results of Univariate testing of Day 14 serum protein levels stratified by clinical outcomes Acute GvHD: Yes (44) vs No (105) **(A)**; Chronic GvHD: Yes (22) vs No (122) **(B)**; Relapse: Yes (44) vs No (105) **(C)**; Survival: Deceased **(D)** (52) vs Alive **(A)** (100) **(D)**. Proteins labelled in red showed a significant difference (p<0.05) by Man Whitney after Benjamini-Hochberg (BH) multiple testing correction. FC estimate is calculated as -1*(median difference between samples from eac distribution). Violin plots of Normalised Protein eXpression (NPX) are shown for the proteins found to be differentially expressed for each clinical outcome. (BH corrected p≤0.01**; p≤0.05*).

We next determined how protein levels at day 14 were associated with subsequent clinical outcomes (n=142). Overview visualisations of the data in reduced dimensional (UMAP) space did not show clear groupings (**Supplementary Figures S1**, **2**) therefore protein levels were specifically assessed, stratifying by binary outome. High levels of 5 proteins in patients who developed AGvHD, with the highest concentrations observed for LIFR and CCL23 (**Figure 5A**). Of note, low levels of LIFR at day 0 (**Figure 1E**) and a large increase in the D0:D14 ratio was also seen in patients with AGvHD (**Figure 4A**). Flt3L expression was lower in patients with AGvHD (**Figure 5A**) whilst the only association with CGvHD was seen for CASP8 where higher levels were associated with increased risk of CGvHD (**Figure 5B**).

A low serum concentration of 5 proteins, IL-6, IL-17C, IL-2, MMP-10 and CXCL10, was seen in patients with subsequent disease relapse and a similar association has been observed with suppressed D14:D0 ratio for three of these, IL-17C, MMP-10 and CXCL10 (**Figure 5C**). 10 proteins were elevated in patients who subsequently died with the most marked effects observed for CDCP1, PDL1, and IL-10 (**Figure 5D**) and it is notable that differential expression of these proteins had not been observed on the day of transplant (D0).

### Systemic inflammatory protein concentrations at day 14 are independently associative of subsequent clinical outcome

Statistical models were next used to investigate the association between protein concentration at day 14 and the time to clinical events during median 3 years of follow up (CI 2-3.9). To reduce the number of proteins included in subsequent statistical models thus reducing type I error rates, only proteins identified as having outcome association from the previous differential expression analysis were included. Univariate estimates for all events (**Supplementary Table 1**) and complete multivariate estimates, including adjustments for clinical covariates for each event, were determined (**Supplementary Tables 2**-**7**). A range of clinical variables were associated with outcome (**Supplementary Tables 3**-**5**). 29% (CI 0.21 - 0.37) of patients developed AGvHD (grade II-IV) whilst 20% developed CGvHD (CI 0.21 - 0.38). The relapse rate was 29% (CI 0.21 - 0.37), the probability of relapse-related mortality (RM) was 18% (CI 0.10 - 0.25) and non-relapse mortality (NRM) rate was 20% (CI 0.12 - 0.28). The probability of surviving without GvHD or relapse (GvHD-free, relapse-free survival (GRFS)) was 28% (CI 0.21-0.37) whilst overall survival (OS) was 62% at three years (CI 0.62 - 0.79). Sex and diagnosis distribution of the cohort had little impact on the results however Myeloid diagnosis was associated (p=0.01;HR=8.35) with increased relapse related mortality following multivariate testing (**Supplementary Tables 1**, **4**).

**Figure 6:**
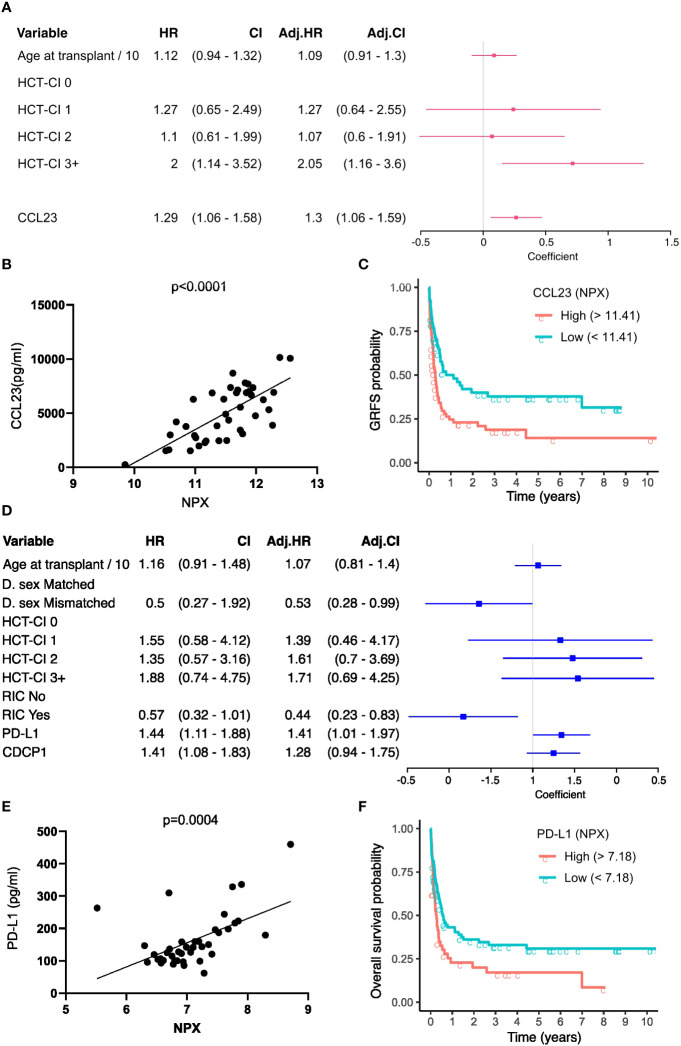
GvHD-free, relapse-free survival (GRFS) and Overall survival associated serum proteins at Day-14. **(A)** Univariate (HR) and multivariate hazard ratios (adj. HR) with a 95% confidence interval (CI) based on the CoxPH model of GRFS. Regression coefficients are visualised as a forest plot (95% CI). **(B)** Correlation of relatively quantified Olink Normalised protein expression (NPX) data with absolute quantification of protein expression by ELISA in pg/ml for CCL23 (n=34). **(C)** Kaplan Meier curves of GRFS probability in patients stratified by high and low day 14 absolute CCL23 concentration. **(D)** Univariate and multivariate HR based on the CoxPH model of Overall Survival (OS). **(E)** Correlation of NPX with absolute quantification of protein expression by ELISA for PD-L1. **(F)** Kaplan Meier curves of OS probability in patients stratified by day 14 absolute PD-L1 concentration. CoxPH models adjusted for variables retained after LASSO selection. CCL23 and PD-L1 optimal concentration cutoff determined by maximally selected rank statistics.

Multivariate time-to-event analysis to account for competing risks was used to determine how serum protein concentration at day 14 correlated with the rate of subsequent clinical events (**Table 2**). Levels of CCL23 were associated with increased AGvHD (HR_SD_ 1.66, CI 1.12 - 2.46; P 0.01) and lower rates of GRFS by univariate and multivariate analysis (HR 1.3; CI 1.06 - 1.59; P 0.01) (**Figure 6A**; **Supplementary Table 2**). For potential clinical utility, it was important to calculate absolute serum concentrations of CCL23 and prove correlation with Olink NPX readout (**Figure 6B**). Indeed, stratifying by CCL23 concentration showed GRFS was 40% at 2 years in patients with low CCL23 concentration and 23% in those with high CCL23 (**Figure 6C**). CXCL10 concentration was protective against relapse (HR_SD_ 0.66; CI 0.48 - 0.9; P 0.01) (**Supplementary Table 7**) whilst elevated CDCP1 markedly increased risk of relapse mortality (HR_SD_ 2.41; CI 1.27 - 4.56; P 0.01). IL-6 reduced this risk (HR_SD_ 0.47; CI 0.22 - 1; P 0.05) but also increased non-relapse mortality (HR_SD_ 1.5; CI 1 - 2.23; P 0.05). The only protein associated with lower overall survival was PD-L1 where high levels were associated with increased risk of mortality (HR 1.41; CI 1.01 - 1.97; P 0.05) (**Figure 6D**; **Supplementary Table 3**). Olink NPX readout for PD-L1 correlates with absolute concentration (**Figure 6E**) and stratifying by PD-L1 showed survival at 2 years was 20% for patients with high concentrations compared to 36% in those with low PD-L1 (**Figure 6F**).

**Table 2:** Multivariate competing risk analysis of protein concentration at Day 14 in relation to rate of Relapse and GvHD outcomes.

	Relapse-related mortality			Non-relapsemortality			AGvHD			CGvHD		
	Adj. HR_SD_	Adj.CI	p	Adj. HR_SD_	Adj.Cl	p	Adj. HR_SD_	Adj.Cl	p	Adj. HR_SD_	Adj.CI	p
CCL23	1.94	(0.78 4.87)	0.16	1.11	(0.62 1.97)	0.73	1.66	(1.12 2.46)	0.01	1.14	(0.79 1.64)	0.5
CDCP1	2.41	(1.27 4.56)	0.01	1.26	(0.82 1.94)	0.29	0.93	(0.64 1.35)	0.7	0.9	(0.59 1.38)	0.64
CSF-1	0.93	(0.34 2.54)	0.88	1.57	(0.95 2.6)	0.08	0.95	(0.6 1.51)	0.83	0.7	(0.39 1.24)	0.22
CXCL10	0,57	(0.32 1.02)	0.06	1.29	(0.76 2.2)	0.34	0.89	(0.59 1.35)	0.6	0.96	(0.64 1.42)	0.83
IL6	0.47	(0.22 1)	0.05	1.5	(1 2.23)	0.05	1.13	(0.77 1.65)	0.54	1.14	(0.75 1.71)	0.54
LIF-R	1.48	(0.66 3.3)	0.35	0.47	(0.28 0.77)	0						
PD-L1	0.6	(0.24 1.5)	0.28	1.35	(0.71 2.55)	0.36	0.87	(0.5 1.52)	0.62	1.5	(0.8 2.82)	0.2
TNFRSF9	2.39	(0.81 7.03)	0.11	0.75	(0.4 1.41)	0.37	0.99	(0.69 1.42)	0.95	0.85	(0.53 1.36)	0.5

Multivariate subdistribution hazard ratios (adj. HR_SD_) with a 95% confidence interval (CI) based on FG models of Relapse related mortality, Non-relapse mortality, acute GvHD and chronic GvHD. Models adjusted for variables retained after LASSO selection, excluding any variable in violation of proportional hazards assumption.

These findings show that serum concentrations at day 14 can provide strong value for independent prediction of subsequent clinical outcome.

## Discussion

Several studies have shown that the concentration of specific serum proteins can help to predict subsequent clinical outcome following HSCT. In this study we show that measurement of inflammation-associated proteins at both day 0 and day 14 can provide additional novel prognostic insights. These observations uncover insights into determinants of alloreactive immunity and could help to guide personalised patient care.

Low levels of seven proteins on the day of stem cell infusion were associated with risk of subsequent GvHD whilst high values of seven different proteins were seen in patients who subsequently suffered disease relapse. These proteins included several members of the cytokine receptor family, such as IL-15R, IL-18R and IL-22R, and may reflect their importance in homeostatic proliferation (30). These results indicate that a low inflammatory status at the time of transplant is associated with clinical evidence of strong subsequent alloreactive immunity. This is of note as low levels of inflammatory markers at the time of immune checkpoint inhibition (ICI) also correlate with improved long term survival (31). Together these indicate that the baseline inflammatory profile of patients at the time of cancer immunotherapy is a direct correlate of subsequent clinical outcome. In relation to the potential clinical utility of this finding, it is possible that this profile is immutable and not amenable to modulation. However, anti-inflammatory agents have been shown to increase the therapeutic response to ICI and CAR-T in mice (32, 33) and their use prior to transplantation might therefore be assessed.

Elevated levels of Flt3L at baseline were associated with superior long term outcome and as values increase reciprocally with the intensity of transplant conditioning and clearance of Flt3+ AML (29), both of these factors could contribute to this observation. Low concentrations of CCL7 (MCP3), a leucocyte chemoattractant, were also associated with reduced overall survival (**Figure 1H**). Elevated baseline levels have been associated with increased risk of graft failure after allo-HSCT (34) although the mechanisms that underlie its prognostic impact remain uncertain.

Protein concentrations were also measured at day 14 to allow an assessment of relative transition during the first two weeks following transplantation. The concentration of many proteins rose during this period and likely reflects transplant conditioning and initiation of the alloreactive immune response. A striking observation was that the levels of 37 proteins at day 14t were strongly correlated with the age of the patient. In contrast, Flt-3 was the only protein for which lower concentrations were seen in older patients. Only 5 inflammatory proteins were elevated at Day 0 in older patients and this increment over 14 days therefore reveals enhanced transplant-induced inflammation in older people. This is likely to represent an immunological determinant of the established correlate of patient age with impaired transplant outcome.

The trajectory of change between day 0 and day 14 proved to be a stronger indicator of clinical outcome than absolute values at either timepoint. An increase in 11 proteins associated with risk of GvHD whilst a decrease, or muted increase, in 18 proteins was linked to disease relapse.

Overall, these findings indicate that the temporal dynamics of inflammatory markers in the first two weeks after transplantation are a strong correlate of subsequent clinical outcome. In particular, patients with relatively low systemic inflammatory markers at the time of stem cell infusion, and who subsequently develop a strong inflammatory response in the subsequent 2 weeks, are at increased risk of GVHD. However, as alloreactive immunity helps to control the underlying tumour, an inverse correlation was also observed in relation to disease relapse. In particular, patients entering transplantation within higher inflammatory markers, or in whom subsequent inflammatory markers decreased or increased only modestly during the subsequent 2 weeks, were at high risk of disease relapse.

Although strong inflammatory responses were associated with decreased risk of disease relapse, they had less impact on survival. These findings indicate the delicate balance that exists between inflammatory responses which act to control disease relapse but also promote GvHD, tissue damage and transplant-related mortality. An important determinant here will be the impact of patient age, that acts both to increase frailty and demographic risk but also increases baseline inflammatory responses due to ‘inflamm-ageing’.

Multivariate analysis allowed identification of protein concentrations at day 14 independently associative of subsequent clinical outcome. CCL23 concentration associated with occurrence of acute GvHD and is likely to reflect its role as a strong chemoattractant for T cells and monocytes. Similarly, CXCL10 concentration was a marker of subsequent disease relapse and is an important regulator of the alloreactive immune response (35). Finally, high levels of PD-L1 were predictive of increased risk of mortality and this concurs with previous studies showing this relationship at later timepoints (36).

Whilst previous reports have identified elevated MCP-1 at day 30 post-transplant as a predictor of subsequent relapse (37), our study did not reveal a significant correlation between MCP-1 levels at day 0 or day 14 and clinical outcomes. This suggests that MCP-1 may not serve as an early indicator of adverse clinical outcomes. Furthermore, despite the established link between IL-6 and AGVHD severity (6), our study did not identify significant univariate associations for IL-6. Conversely, IL-33, known to increase in AGVHD (38), did not exhibit such associations but demonstrated reduced levels in CGVHD. These findings provide nuanced insights into the intricate roles of MCP-1, IL-6, and IL-33 in post-transplant scenarios, further highlighting the complexity and importance of studying kinetics of these cytokines in the post-transplant setting.

Limitations of our study include the lack of inclusion of ST2 and epithelial markers which are known predictive markers for GvHD (4, 5). Additionally, variables such as HLA mismatch could not be included due to large class imbalance (n=13 (Yes) vs n=104 (No)). ATG vs no ATG had to be omitted from multivariate models as it violated proportional hazard assumptions. Furthermore, the relatively small nature of the cohort is such that findings should be considered for future validation in other cohorts.

The observation that inflammatory protein concentrations at very early timepoints are associated with clinical events that occur many months later adds further support to the concept that the clinical outcome of transplantation is strongly influenced by immunological priming within days of stem cell infusion. Our findings suggest candidate predictive serum protein biomarkers for further study with potential relevance to the clinical management of patients following transplantation. The measurement of protein concentration at day 0 and day 14 is feasible within the care plan and their predictive value deserves future assessment as a potential guide to optimize personalised care for patients undergoing this challenging procedure.

## Data availability statement

The original contributions presented in the study are included in the article/**Supplementary Material**. Further inquiries can be directed to the corresponding author.

## Ethics statement

The studies involving humans were approved by NRES Committee West Midlands – South Birmingham (BOOST 15/WM/0194). The studies were conducted in accordance with the local legislation and institutional requirements. The participants provided their written informed consent to participate in this study.

## Author contributions

KV: Conceptualization, Data curation, Formal analysis, Investigation, Methodology, Project administration, Validation, Visualization, Writing – original draft, Writing – review & editing. WC: Formal analysis, Investigation, Methodology, Software, Visualization, Writing – original draft, Writing – review & editing. DG: Formal analysis, Investigation, Methodology, Software, Visualization, Writing – original draft. CS: Data curation, Investigation, Methodology, Project administration, Validation, Writing – review & editing. RM: Data curation, Project administration, Resources, Writing – review & editing. JN: Project administration, Resources, Writing – review & editing. JZ: Conceptualization, Methodology, Project administration, Writing – review & editing. FK: Data curation, Formal analysis, Resources, Writing – review & editing. PM: Conceptualization, Formal analysis, Funding acquisition, Resources, Supervision, Writing – original draft, writing – review & editing.
