# Supplementary material for: Early inflammatory markers as prognostic indicators following allogeneic stem cell transplantation
Source: Front Immunol. 2024 Jan 3;14:1332777. doi: 10.3389/fimmu.2023.1332777 (PMC10791949; doi:10.3389/fimmu.2023.1332777)
Supplement: Supplementary file 1 [file DataSheet_1.pdf]

Supplementary Materials

Supplementary Figure S1. Overview of Day 14 data in reduced dimensional space. UMAP of D14 sample NPX data overlaid with Diagnosis and clinical outcomes Survival, AGvHD, CGvHD and Relapse.

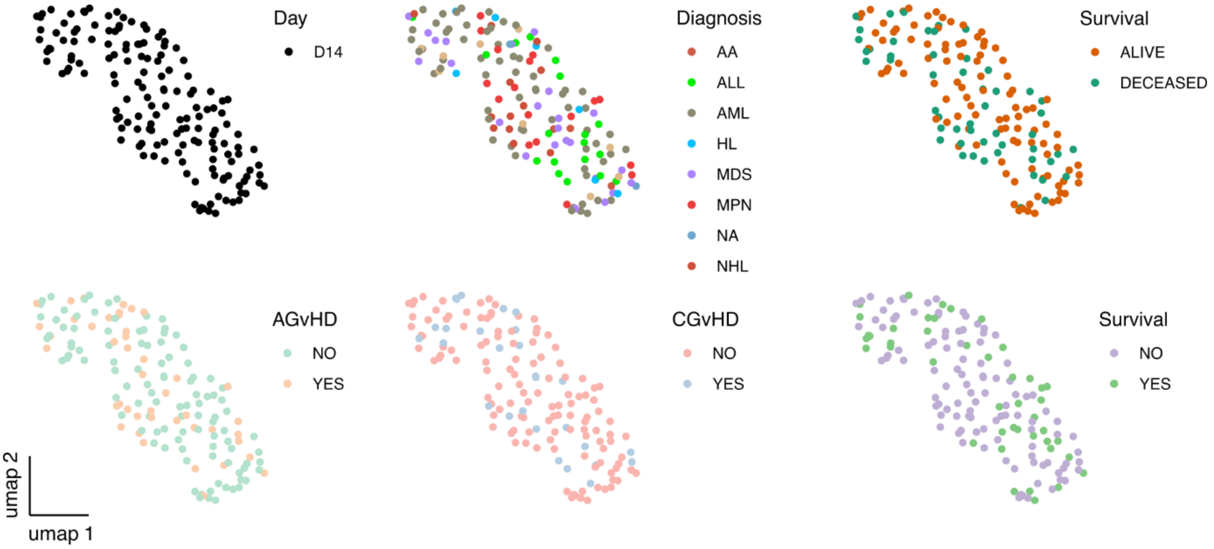

Supplementary Figure S2. Overview of 92 proteins serum expression at day 14 post SCT in reduced dimensional space. UMAP of 154 D14 sample NPX data overlaid with serum protein NPX.

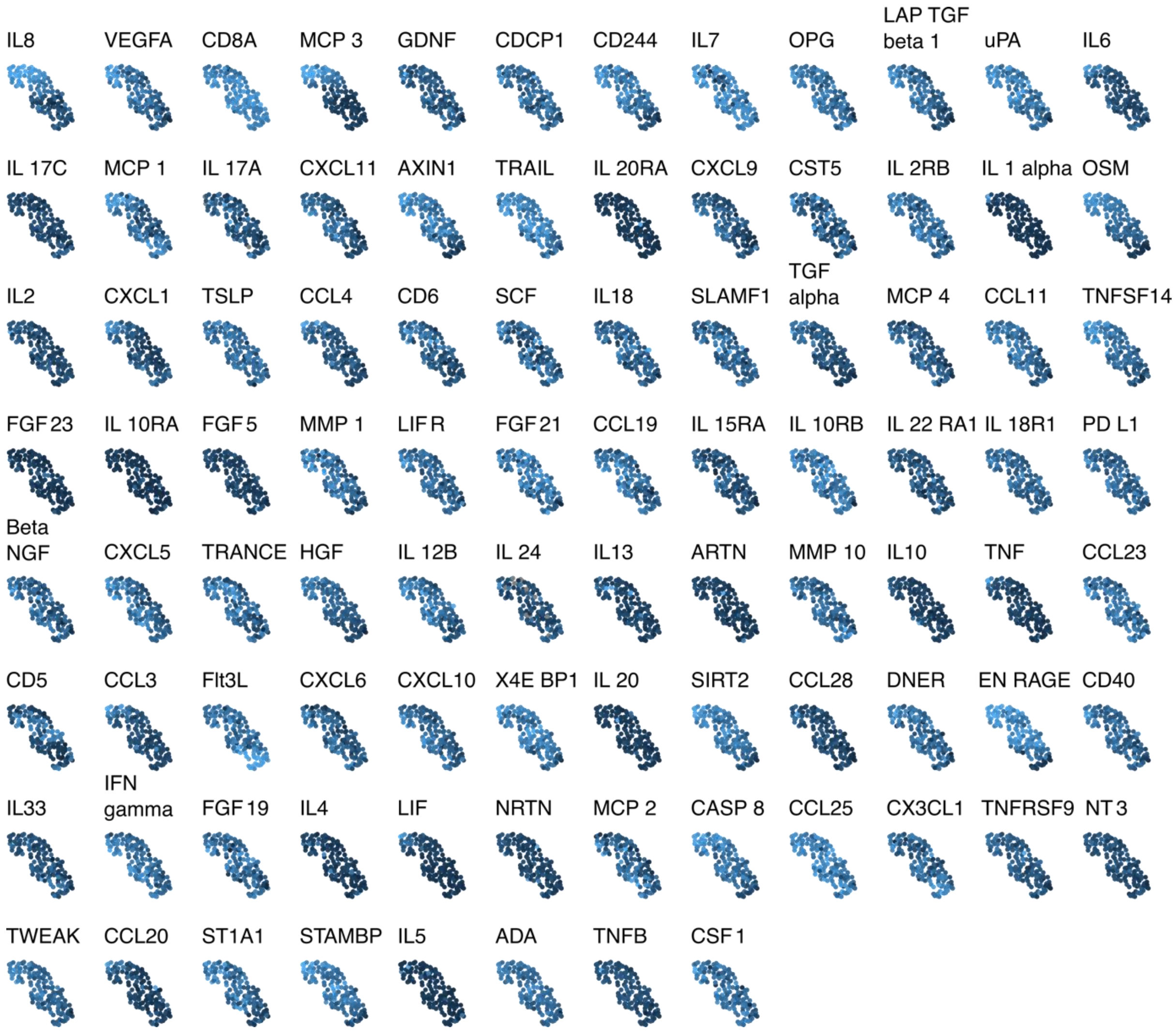

Supplementary Figure S3. Correlation of relatively quantified Olink Normalised protein expression (NPX) data with absolute quantification of protein expression by ELISA in pg/ml for selected proteins. (A) PD-L1 (B) LIFR (C)FLt3L (D) CCL23 (E) IL-6 (F) CDCP1

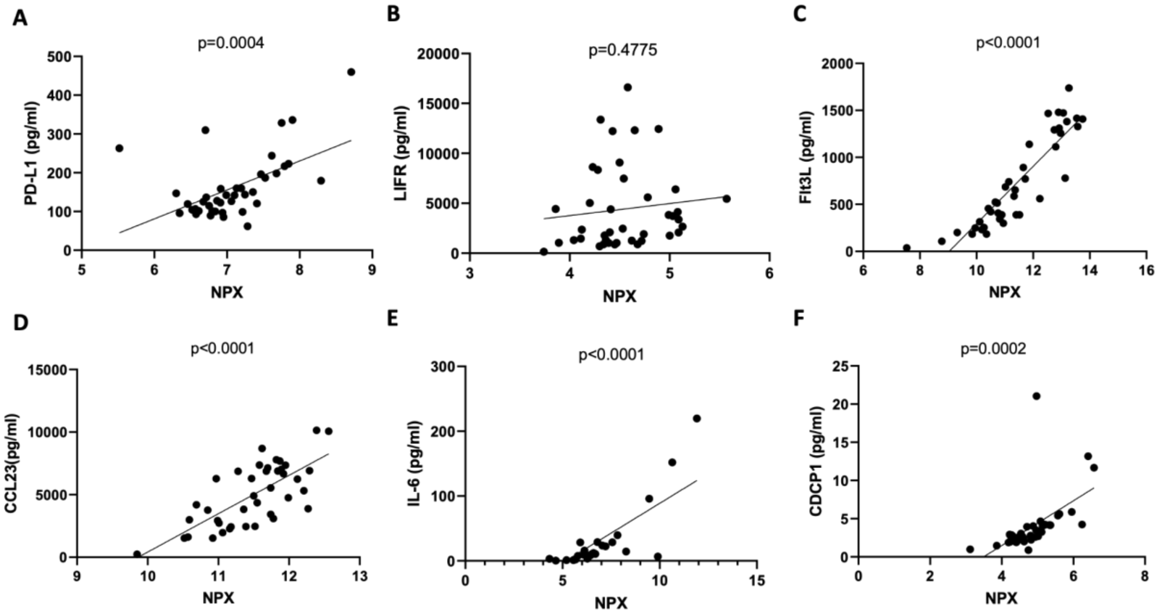

Supplementary Table 1 – Univariate regression estimates for all end-points

(attached in excel)

Supplementary Table 2 – Multivariate regression estimates for GRFS

	GvHD-free, relapse-free survival			
**Variable**	**Adj.HR**	**Adj.CI**	**P val.**	**PH test**
Age at transplant / 10	1.09	(0.91 - 1.3)	0.34	0.519
HCT-CI 0				0.962
HCT-CI 1	1.27	(0.64 - 2.55)	0.50	0.962
HCT-CI 2	1.07	(0.6 - 1.91)	0.81	0.962
HCT-CI 3+	2.05	(1.16 - 3.6)	0.02	0.962
CCL23	1.3	(1.06 - 1.59)	0.01	0.632

Supplementary Table 3 – Multivariate regression estimates for OS

	Overall survival			
**Variable**	**Adj.HR**	**Adj.CI**	**P val.**	**PH test**
Age at transplant / 10	1.07	(0.81 - 1.4)	0.65	0.81
D. sex Matched				0.257
D. sex Mismatched	0.53	(0.28 - 0.99)	0.06	0.257
HCT-CI 0				0.729
HCT-CI 1	1.39	(0.46 - 4.17)	0.56	0.729
HCT-CI 2	1.61	(0.7 - 3.69)	0.27	0.729
HCT-CI 3+	1.71	(0.69 - 4.25)	0.26	0.729
RIC No				0.45
RIC Yes	0.44	(0.23 - 0.83)	0.02	0.45
PD-L1	1.41	(1.01 - 1.97)	0.05	0.623
CDCP1	1.28	(0.94 - 1.75)	0.13	0.614

Supplementary Table 4 – Multivariate regression estimates for RM and NRM

	Relapse-related mortality				Non-relapse mortality			
Variable	Adj. HR (SD)	Adj.CI	P val.	PH test	Adj. HR (SD)	Adj.CI	P val.	PH test
Age at transplant / 10	1.19	(0.69 - 2.06)	0.54	0.10	1.11	(0.69 - 1.8)	0.66	0.40
CD34+ dose	1.02	(0.82 - 1.26)	0.88	0.26	1.24	(1.02 - 1.49)	0.03	0.30
Diagnosis Lymphoid								
Diagnosis Myeloid	8.35	(1.87 - 37.32)	0.01	0.32	0.27	(0.1 - 0.73)	0.01	0.86
HCT-CI 0								
HCT-CI 1	1.24	(0.15 - 10.16)	0.85	0.39	0.83	(0.11 - 6.24)	0.86	0.50
HCT-CI 2	2.22	(0.37 - 13.42)	0.39	0.33	1.22	(0.31 - 4.87)	0.77	0.34
HCT-CI 3+	0.32	(0.03 - 3.96)	0.39	0.46	4.9	(1.17 - 20.59)	0.04	0.49
P. sex Female								
P. sex Male	1.7	(0.4 - 7.14)	0.47	0.30	0.94	(0.35 - 2.49)	0.90	0.49
TBI No								
TBI Yes	3.98	(0.51 - 30.76)	0.19	0.28	1.35	(0.39 - 4.7)	0.63	0.70
CCL23	1.94	(0.78 - 4.87)	0.16	0.28	1.11	(0.62 - 1.97)	0.73	0.92
CDCP1	2.41	(1.27 - 4.56)	0.01	0.50	1.26	(0.82 - 1.94)	0.29	0.60
CSF-1	0.93	(0.34 - 2.54)	0.88	0.12	1.57	(0.95 - 2.6)	0.08	0.09
CXCL10	0.57	(0.32 - 1.02)	0.06	0.08	1.29	(0.76 - 2.2)	0.34	0.97
IL6	0.47	(0.22 - 1)	0.05	0.07	1.5	(1 - 2.23)	0.05	0.46
LIF-R	1.48	(0.66 - 3.3)	0.35	0.31	0.47	(0.28 - 0.77)	0.00	0.19
PD-L1	0.6	(0.24 - 1.5)	0.28	0.35	1.35	(0.71 - 2.55)	0.36	0.06
TNFRSF9	2.39	(0.81 - 7.03)	0.11	0.12	0.75	(0.4 - 1.41)	0.37	0.28

Supplementary Table 5 – Multivariate regression estimates for AGvHD and death (any other cause)

	AGvHD				Death (without AGvHD)			
**Variable**	**Adj. HR (SD)**	**Adj.CI**	**P val.**	**PH test**	**Adj. HR (SD)**	**Adj.CI**	**P val.**	**PH test**
Age at transplant / 10	1.46	(1.03 - 2.08)	0.04	0.64	0.93	(0.68 - 1.29)	0.68	0.65
CD34+ dose	1.06	(0.88 - 1.27)	0.55	0.60	1	(0.88 - 1.14)	0.94	0.73
Diagnosis Lymphoid								
Diagnosis Myeloid	0.9	(0.37 - 2.21)	0.81	0.19	0.92	(0.39 - 2.2)	0.86	0.39
HCT-CI 0								
HCT-CI 1	1.22	(0.37 - 3.99)	0.74	0.19	0.75	(0.16 - 3.55)	0.72	0.35
HCT-CI 2	0.83	(0.25 - 2.71)	0.75	0.20	1.08	(0.37 - 3.18)	0.88	0.22
HCT-CI 3+	2.6	(0.97 - 6.95)	0.06	0.38	0.91	(0.24 - 3.37)	0.89	0.10
P. sex Female								
P. sex Male	1.08	(0.53 - 2.17)	0.84	0.91	1.01	(0.41 - 2.5)	0.98	0.59
TBI No								
TBI Yes	1.48	(0.66 - 3.34)	0.34	0.65	1.89	(0.54 - 6.6)	0.32	0.62
CCL23	1.66	(1.12 - 2.46)	0.01	0.74	0.65	(0.35 - 1.2)	0.17	0.03
CDCP1	0.93	(0.64 - 1.35)	0.70	0.96	1.37	(0.98 - 1.92)	0.07	0.23
CSF-1	0.95	(0.6 - 1.51)	0.83	0.42	1.13	(0.64 - 1.97)	0.68	0.77
CXCL10	0.89	(0.59 - 1.35)	0.60	0.45	1.04	(0.65 - 1.64)	0.88	0.65
IL6	1.13	(0.77 - 1.65)	0.54	0.07	0.89	(0.62 - 1.29)	0.55	0.73
PD-L1	0.87	(0.5 - 1.52)	0.62	0.39	1.19	(0.72 - 1.96)	0.51	0.50
TNFRSF9	0.99	(0.69 - 1.42)	0.95	0.43	1.36	(0.91 - 2.04)	0.13	0.20

Supplementary Table 6 – Multivariate regression estimates for CGvHD and death (any other cause)

	CGvHD				Death (without CGvHD)			
Variable	Adj. HR (SD)	Adj.CI	P val.	PH test	Adj. HR (SD)	Adj.CI	P val.	PH test
Age at transplant / 10	1.03	(0.76 - 1.41)	0.83	0.38	1.02	(0.71 - 1.48)	0.91	0.71
CD34+ dose	1.07	(0.87 - 1.3)	0.53	0.91	1.11	(0.98 - 1.26)	0.10	0.20
Diagnosis Lymphoid								
Diagnosis Myeloid	0.91	(0.35 - 2.33)	0.84	0.26	1	(0.42 - 2.4)	1.00	0.43
HCT-CI 0								
HCT-CI 1	1.01	(0.2 - 5.02)	0.99	0.50	1.03	(0.31 - 3.41)	0.96	0.70
HCT-CI 2	0.94	(0.3 - 2.97)	0.92	0.32	1.05	(0.3 - 3.6)	0.94	0.23
HCT-CI 3+	0.67	(0.17 - 2.64)	0.57	0.44	2.35	(0.89 - 6.19)	0.09	0.38
TBI No								
TBI Yes	1.73	(0.5 - 5.98)	0.38	0.77	1.19	(0.42 - 3.34)	0.74	0.83
CCL23	1.14	(0.79 - 1.64)	0.50	0.58	1.06	(0.65 - 1.72)	0.83	0.60
CDCP1	0.9	(0.59 - 1.38)	0.64	0.12	1.26	(0.88 - 1.81)	0.21	0.85
CSF.1	0.7	(0.39 - 1.24)	0.22	0.18	1.37	(0.83 - 2.26)	0.22	0.39
CXCL10	0.96	(0.64 - 1.42)	0.83	0.12	0.9	(0.6 - 1.35)	0.63	0.49
IL6	1.14	(0.75 - 1.71)	0.54	0.56	1.02	(0.74 - 1.4)	0.90	0.71
PD-L1	1.5	(0.8 - 2.82)	0.20	0.07	1.08	(0.67 - 1.74)	0.74	0.25
TNFRSF9	0.85	(0.53 - 1.36)	0.50	0.11	1.09	(0.52 - 2.25)	0.82	0.40

Supplementary Table 7 – Multivariate regression estimates for relapse and NRM

	Relapse				Non-relapse mortality			
**Variable**	**Adj. HR (SD)**	**Adj.CI**	**P val.**	**PH test**	**Adj. HR (SD)**	**Adj.CI**	**P val.**	**PH test**
Age at transplant / 10	1.04	(0.77 - 1.4)	0.80	0.55	1.13	(0.7 - 1.81)	0.62	0.41
CD34+ dose	1.07	(0.93 - 1.23)	0.35	0.34	1.21	(1 - 1.46)	0.05	0.31
Diagnosis Lymphoid								
Diagnosis Myeloid	2.3	(1 - 5.26)	0.05	0.05	0.24	(0.08 - 0.7)	0.01	0.78
HCT-CI 0								
HCT-CI 1	1.43	(0.36 - 5.57)	0.61	0.41	1	(0.12 - 8.31)	1.00	0.59
HCT-CI 2	1.63	(0.7 - 3.79)	0.26	0.53	1.24	(0.3 - 5.12)	0.77	0.34
HCT-CI 3+	0.55	(0.18 - 1.67)	0.29	0.35	4.62	(1.01 - 21.26)	0.06	0.18
P. sex Female								
P. sex Male	0.78	(0.38 - 1.61)	0.50	0.52	0.95	(0.35 - 2.6)	0.93	0.59
TBI No								
TBI Yes	0.67	(0.21 - 2.16)	0.50	0.11	1.38	(0.42 - 4.55)	0.60	0.48
CCL23	1.42	(0.87 - 2.31)	0.16	0.76	1.16	(0.65 - 2.08)	0.61	0.93
CDCP1	1.29	(0.91 - 1.84)	0.16	0.88	1.26	(0.82 - 1.95)	0.29	0.56
CXCL10	0.66	(0.48 - 0.9)	0.01	0.47	1.49	(0.89 - 2.5)	0.13	0.97
IL6	0.72	(0.5 - 1.04)	0.08	0.37	1.54	(1.04 - 2.28)	0.03	0.47
LIF-R	1.04	(0.73 - 1.48)	0.82	0.83	0.48	(0.28 - 0.82)	0.01	0.16
PD-L1	0.9	(0.49 - 1.65)	0.72	0.67	1.44	(0.79 - 2.63)	0.23	0.07
TNFRSF9	1.15	(0.6 - 2.23)	0.67	0.10	0.86	(0.49 - 1.51)	0.61	0.37
